# Supplementary material for: Genomic interrogation of a MAGIC population highlights genetic factors controlling fiber quality traits in cotton
Source: Commun Biol. 2022 Jan 17;5:60. doi: 10.1038/s42003-022-03022-7 (PMC8764025; doi:10.1038/s42003-022-03022-7)
Supplement: Supplementary file 2 — Supplementary Information [file 42003_2022_3022_MOESM2_ESM.pdf]

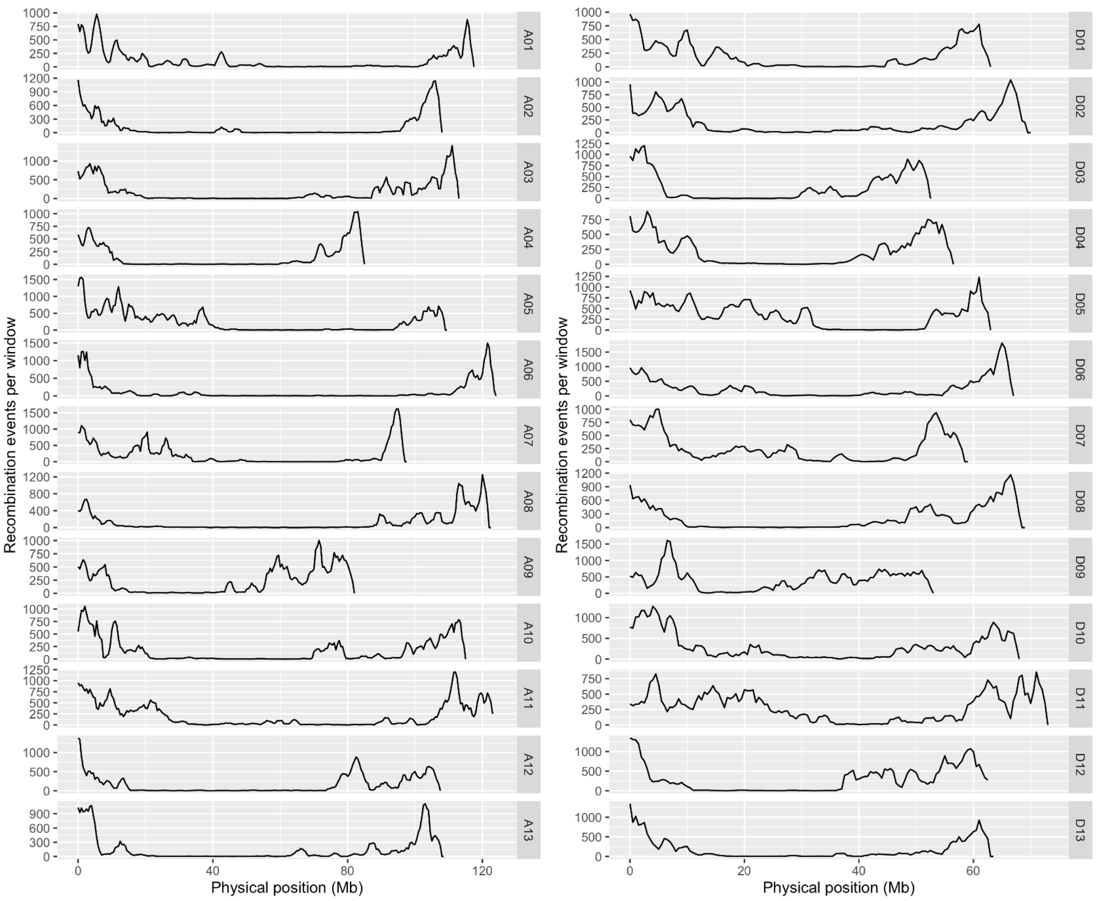

Supplementary Fig. 1 Recombination events for 26 chromosomes. Each estimated point indicates the sum of recombination events in a 2-Mb sliding window across the 550 MAGIC RILs.

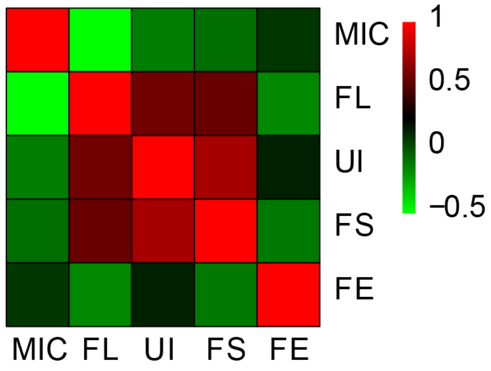

Supplementary Fig. 2 A heatmap of Pearson correlation coefficients over five fiber quality traits. The five traits include elongation (FE), length (FL), strength (FS), uniformity (UI) and micronaire value (MIC).

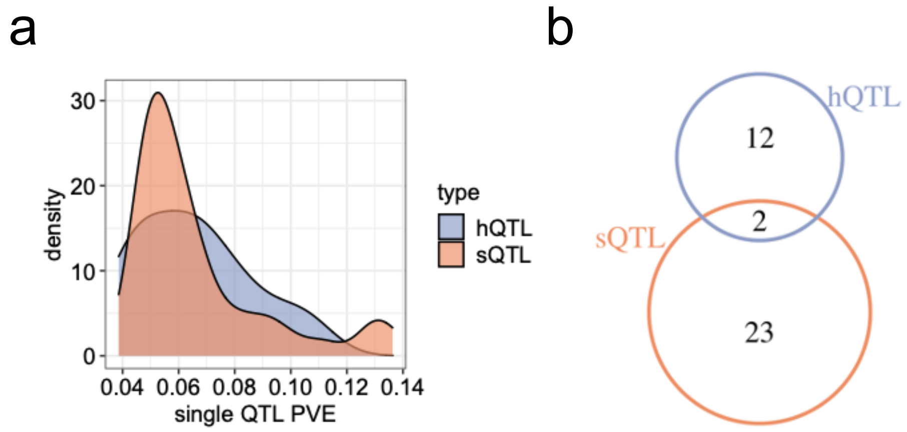

Supplementary Fig. 3 Overview of the two methods of GWAS analysis. a Comparison of phenotypic variance explained (PVE) by single QTL identified by sGWAS vs. hGWAS. **b** Venn diagram of co-localization between sQTL and hQTL, summed over traits.

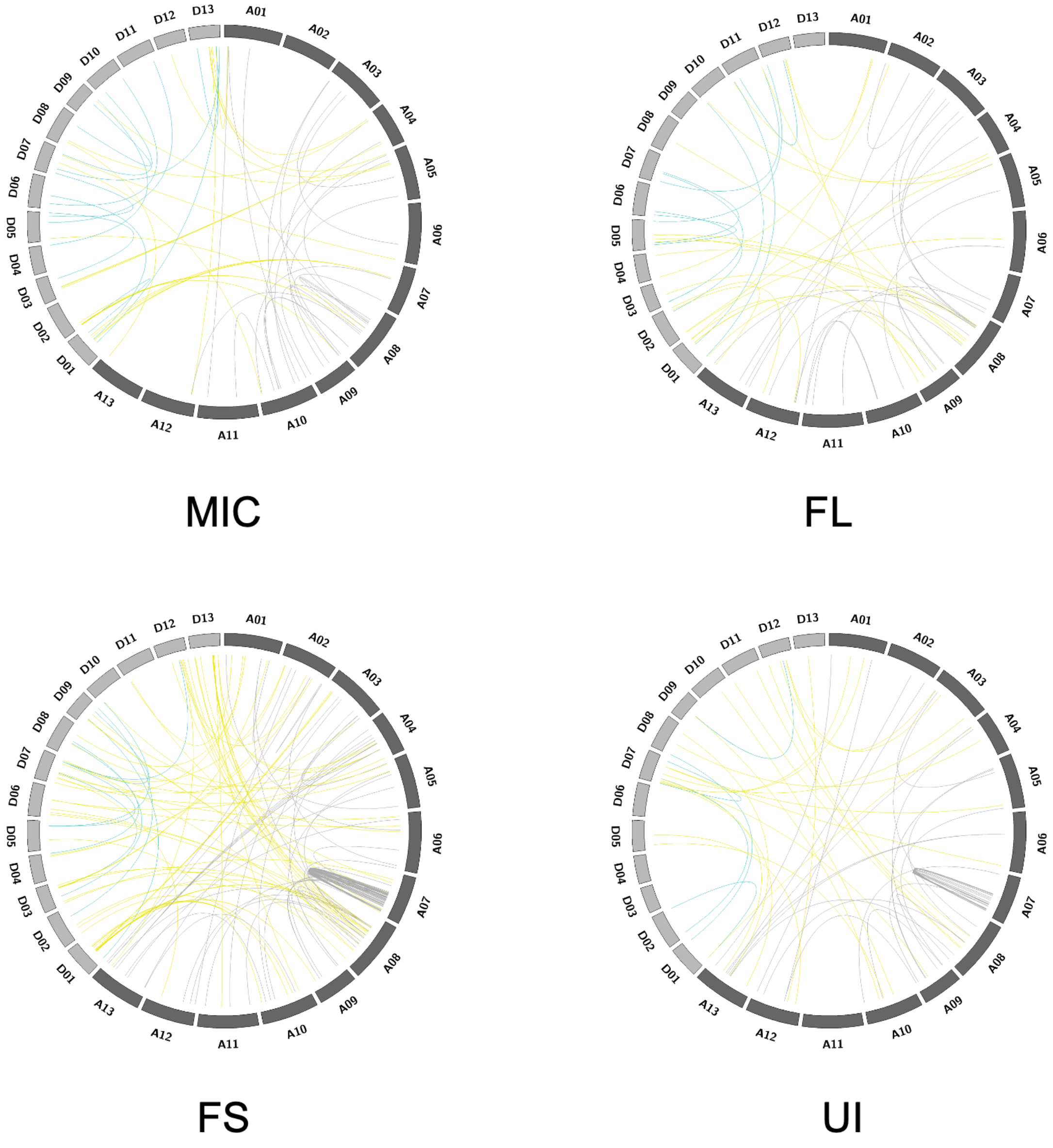

Supplementary Fig. 4 Significant epistasis in trait variance. Continued from Fig 6a. Each circle represents a trait, and each line links an epistatic pair of loci.

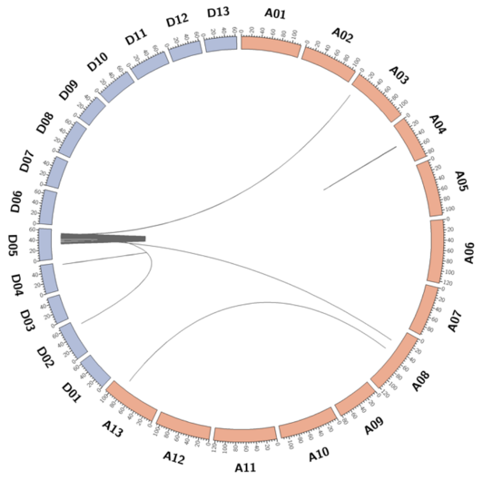

FE

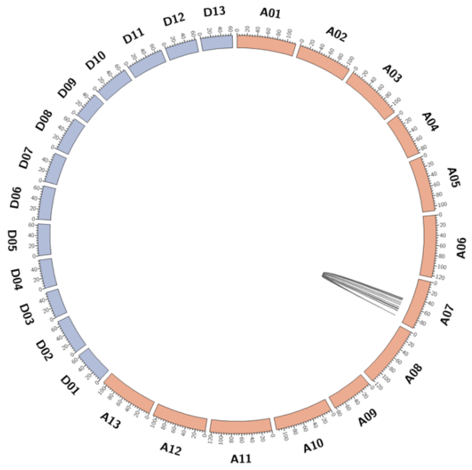

UI

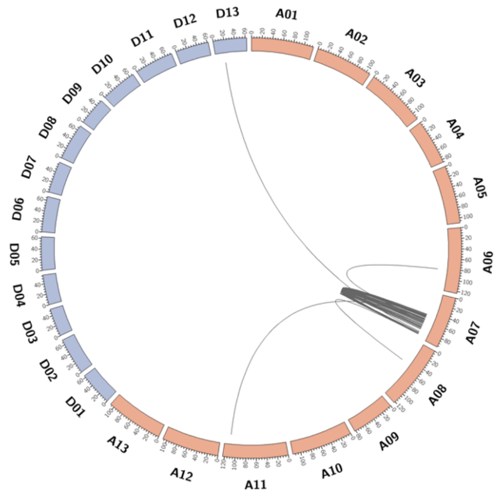

FS

Supplementary Fig. 5 epiQTLs linked with significant loci identified by sGWAS.

The interval of epiQTL was defined as the physical position range delimited by the bin. Traits without these linked epiQTLs are not indicated.

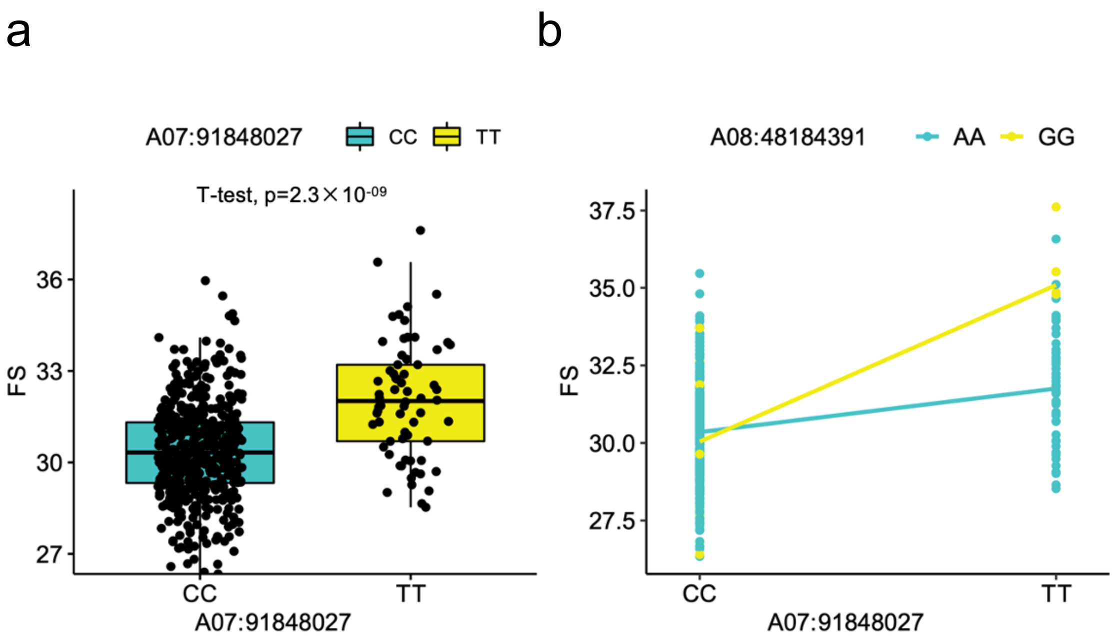

Supplementary Fig. 6 An example of the epistatic effect between a sQTL and other locus. a Boxplot of FS in two genotypes of a significant locus (A07:91848027) identified by sGWAS. **b** Interaction plot for epistasis between A07:91848027 and A08:48184391.

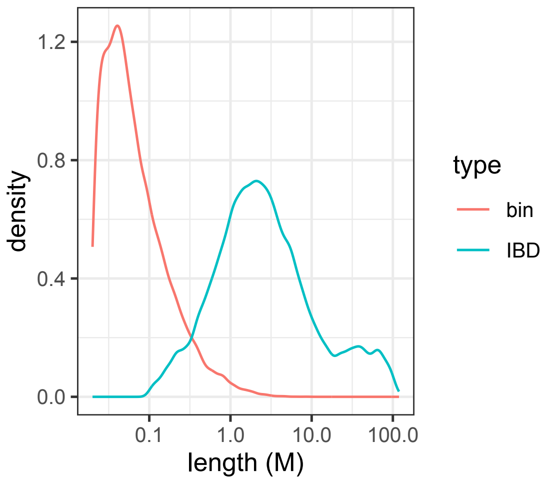

Supplementary Fig. 7 Density plots of the length of IBD segments and bins. The genome of each MAGIC RIL was divided into 18,003 bins based on all identified recombination break points.
