# Supplementary material for: Genomic interrogation of a MAGIC population highlights genetic factors controlling fiber quality traits in cotton
Source: Commun Biol. 2022 Jan 17;5:60. doi: 10.1038/s42003-022-03022-7 (PMC8764025; doi:10.1038/s42003-022-03022-7)
Supplement: Supplementary file 3 — Description of Additional Supplementary Files [file 42003_2022_3022_MOESM3_ESM.pdf]

Description of Additional Supplementary Files

File name: Supplementary Data 1
Description: Summary of categorized SNPs.

File name: Supplementary Data 2
Description: Summary of categorized SNPs in exonic regions.

File name: Supplementary Data 3
Description: IBD information.

File name: Supplementary Data 4
Description: The average number of recombination events per generation per chromosome.

File name: Supplementary Data 5
Description: Information of recombination break points.

File name: Supplementary Data 6
Description: Summary of QTL via sGWAS.

File name: Supplementary Data 7
Description: Genes with nonsynonymous SNPs in sQTL.

File name: Supplementary Data 8
Description: The estimated heritability for each trait and summary of QTL number and their contributions to total PVE.

File name: Supplementary Data 9
Description: Summary of QTL via hGWAS.

File name: Supplementary Data 10
Description: Genes with non-synonymous SNPs in hQTL.

File name: Supplementary Data 11
Description: List of significant epistatic pairs.

File name: Supplementary Data 12
Description: The number and proportion of significant epistatic pairs of different type.

File name: Supplementary Data 13
Description: Information of epiQTLs linked with significant loci identified by sGWAS.

File name: Supplementary Data 14
Description: Bin information across the whole genome.
